# Effectiveness of inspiratory muscle training and multicomponent physical training in patients with post-COVID conditions: a systematic review and meta-analysis

**DOI:** 10.1186/s13643-025-02982-1

**Published:** 2025-11-20

**Authors:** Andresa da Costa Correia, Fernanda Ribeiro, Fábio Ferreira Amorim, Paulo Ricardo Giusti, Maria Stella Peccin, Aline Mizusaki Imoto

**Affiliations:** 1 Escola Superior de Ciências da Saúde (ESCS), Brasília, Federal District Brazil; 2 Centre Intersectorialen Santé Durable, Département de Sciences de La Santé, University of Québec at Chicoutimi, Saguenay, Canada; 3 Universidade Federal de São Paulo (UNIFESP), São Paulo, SP Brazil; 4 Escola de Saúde Pública Do Distrito Federal, Brasília, Federal District Brazil

**Keywords:** Post-COVID condition, Fatigue, Dyspnea, Exercise training, Physical functioning

## Abstract

**Background:**

There is evidence that fatigue and dyspnea are among the most frequently reported symptoms of post-COVID condition. Therefore, several studies have investigated respiratory muscle or global peripheral muscle training as strategies to manage those symptoms. Despite evidence of potential benefits, conflicting results persist due to the heterogeneity of rehabilitation protocols and assessment tools. Thereby, the objective of this systematic review was to evaluate the effectiveness of inspiratory muscle training and multicomponent physical training in adults with dyspnea and fatigue for at least 12 weeks after COVID-19.

**Method:**

A search was conducted in September 2024, in the Cochrane Library (Cochrane Central Register of Controlled Trials), EMBASE, PubMed/MEDLINE, PEDro, Lilacs/BVS, Web of Science, Scopus, and Epistemonikos databases. The inclusion criteria were randomized clinical trials published in any language that evaluated the effectiveness of inspiratory muscle training and multicomponent physical training to improve fatigue, dyspnea, and/or physical function in adults with persistent post-COVID symptoms. The risk of bias of the included studies and the certainty of the evidence were assessed using the RoB 2 and GRADE tools, respectively.

**Results:**

After the screening process, seven randomized clinical trials were included. The total number of participants included in the studies was 449. Inspiratory muscle training significantly improved inspiratory muscle strength (maximal inspiratory pressure) (MD = 22.70; 95% CI: 13.78 to 31.62), and cardiopulmonary capacity ($\dot{V}$ O_2max_) (MD = 4.49; 95% CI: 3.35 to 5.62). Multicomponent physical training significantly improved the upper and lower body muscle strength through the handgrip strength (MD = 3.05; 95% CI: 1.68 to 4.42), sit-to-stand test (MD = 3.55; 95% CI: 1.61 to 5.49), and timed up and go test (MD = − 1.13; 95% CI: − 1.49 to − 0.77) and the physical functioning were assessed through post-COVID-19 functional scale (MD = − 0.64; 95% CI: − 1.13 to − 0.16) and physical aspects through SF-12 and SF-36 (SMD = 0.72; 95% CI: 0.29 to 1.15). No adverse events were reported among participants in the physical training group, and treatment adherence ranged from 78 to 100%.

**Conclusion:**

Inspiratory muscle training improved cardiorespiratory outcomes, while multicomponent physical training improved muscle strength, physical functioning, and fatigue. Both types of training improve physical functioning. The certainty of evidence for the outcomes evaluated was low.

**Systematic review registration:**

PROSPERO (CRD42023451057).

**Supplementary Information:**

The online version contains supplementary material available at 10.1186/s13643-025-02982-1.

## Background

The year 2020 was marked by the onset of the COVID-19 pandemic, a respiratory syndrome of variable intensity caused by the SARS-CoV-2 coronavirus with high transmissibility and primarily affecting the respiratory tract, but may also cause multisystemic impairment [1].

While people fully recover after the acute phase of COVID-19 [2], an increasing proportion develop persistent sequelae that may adversely affect quality of life [3]. Moreover, Fiore et al. [4] reported that musculoskeletal pain may be associated with increased inflammatory mediators, thereby exacerbating physical fatigue and further impairing quality of life.

Systematic reviews demonstrated that fatigue and dyspnea are the most reported symptoms by patients, regardless of the severity of SARS-CoV-2 infection in the acute phase [3, 5–8]. Female gender, advanced age, comorbidity, and severe clinical condition in the acute phase of the disease were identified as probable risk factors for post-COVID conditions (PCC) [6, 7].

In our study, the term “post-COVID condition” was preferred over several alternatives to describe persistent symptoms post-COVID [1, 9, 10]. The increasing proportion of patients with chronic symptoms after COVID-19 was sufficiently significant that the World Health Organization (WHO) recommended the use of the codes from the 10th Revision of the International Statistical Classification of Diseases and Related Health Problems (ICD-10), specifically U09.9 for this condition. Diagnosis is based on the persistence of signs and symptoms that develop during or after a confirmed SARS-CoV-2 infection, present for more than 12 weeks, and not attributable to other diagnoses [11].

There is evidence that fatigue and dyspnea are among the most frequently reported symptoms of PCC, and fatigue management is an essential part of the rehabilitation plan [11, 12]. Studies considering patients' and experts' reports suggest that the overall benefits of the intervention substantially outweigh its disadvantages [13, 14].

Exercise has been shown to be an effective non-pharmacological therapy for various chronic diseases, providing benefits to the cardiovascular, respiratory, musculoskeletal, metabolic, and mental systems [15]. The exercise program strategies to manage the most prevalent symptoms in PCC may involve only the respiratory muscles, such as inspiratory muscle training (IMT), or training of the global peripheral musculature, combining different types of exercises, including aerobic, strength, endurance, and muscle flexibility, for example. The training program that combines at least three types of exercises in a single training session is multicomponent physical training (MPT) [16].

However, randomized controlled trials (RCTs) remain scarce or are often evaluated in systematic reviews alongside studies that are less suited to demonstrating the effectiveness of interventions. Despite evidence of potential benefits, conflicting results persist due to the heterogeneity of rehabilitation protocols and assessment tools [17, 18]. This systematic review was conducted to evaluate the effectiveness of inspiratory muscle training and multicomponent physical training in adults with dyspnea and fatigue persisting for at least 12 weeks after COVID-19. These symptoms are the primary targets of rehabilitation exercise training programs, regardless of whether the intervention focuses on respiratory or limb muscle training.

## Methods

This protocol was registered in the Prospective International Registry of Systematic Reviews (PROSPERO) under CRD42023451057 [19]. The methodology was based on the Cochrane Collaboration Handbook [20], and for the reporting, followed the Preferred Reporting Items for Systematic Reviews and Meta-Analyses (PRISMA) guidelines [21].

### Inclusion criteria

The study population was adults with signs and symptoms that developed during or after a confirmed SARS-CoV-2 infection, persisted for more than 12 weeks, and were not attributable to alternative diagnoses [13]. Persistent symptoms should be compatible with fatigue, dyspnea, and impaired physical functioning. Studies conducted with patients diagnosed with myalgic encephalomyelitis/chronic fatigue syndrome (ME/CFS) were excluded. Inspiratory muscle training, MPT, or their combination were considered interventions. The authors recognize IMT as exercises aimed at strengthening the inspiratory muscles, performed against inspiratory resistance imposed by a loading device, according to the percentage of maximal inspiratory pressure (MIP) measured previously [22–24].

Multicomponent physical training was defined as a combination of resistance and muscle strength physical exercises for the upper and lower limbs, performed at variable intensities, and potentially associated with aerobic and flexibility exercises [16, 25, 26], following a protocol similar to that adopted in pulmonary rehabilitation. The control Group was considered to have no intervention or to follow the WHO recommendations for self-management after COVID-19-related diseases [27].

The primary outcomes considered for this systematic review were pulmonary function tests, cardiopulmonary capacity, upper and lower limb muscle strength, perception of fatigue, physical functioning, and perception of dyspnea. Secondary outcomes included treatment adherence and adverse events.

The type of study was RCTs published in any language that evaluated the effectiveness of inspiratory muscle training and multicomponent physical training to improve fatigue, dyspnea, and/or physical function. RCTs with adult populations restricted to one type of professional class, post hoc RCT studies, ongoing RCT protocols, and RCTs with missing data on the duration of fatigue symptoms and/or dyspnea in the PCC were excluded.

### Exclusion criteria

The exclusion criteria were studies evaluating rehabilitation exercise programs that lasted less than 6 weeks or were conducted at a frequency of fewer than two sessions per week. Additionally, studies with participants diagnosed with ME/CFS and those who underwent rehabilitation for physical fatigue after COVID-19 with relevant symptoms for less than 3 months were also excluded.

#### Search strategy

A systematic search of peer-reviewed articles was conducted across the following databases: Cochrane Library (Cochrane Central Register of Controlled Trials), EMBASE, PubMed/MEDLINE, PEDro, Lilacs/BVS, Web of Science, Scopus, andEpistemonikos. The search period spanned from the database’s establishment to September 2024. In the search, terms previously identified in Descriptors in Health Sciences (DeCS) and Medical Subject Headings (MeSH) were used, as well as their respective synonyms, to include the largest number of relevant studies. The search terms used as references were “post-acute COVID-19 syndrome” and “rehabilitation.” To refine the search, the Boolean operators OR and AND combined the related terms and their “entry terms.” The strategy for each database is described in Supplementary Material 1. The search for unpublished studies was conducted in MedRxiv (https://www.medrxiv.org/).

#### Study selection

All articles found by the searches were organized in the Zotero Software. Then, the Covidence Platform for Systematic Review, recommended by the Cochrane Collaboration Handbook, was used to select, screen, and extract data from the studies.

Two reviewers (ACC and PRG) independently selected the studies. The Covidence Platform provides an interface for each reviewer, indicating which studies present divergences that the reviewers agree to resolve consensually.

Initially, titles and abstracts were screened. Disagreements between reviewers regarding the inclusion or exclusion of studies were resolved through discussion until a consensus was achieved. Subsequently, the full texts were assessed, and the final set of studies to be included in the review was determined.

Conflicts were resolved after discussion with a third reviewer (AMI). Studies that did not meet the inclusion criteria were excluded, and the reasons for this decision were recorded in Supplementary Material 2.

The eligible articles were included in the methodological quality assessment stage, using the Cochrane Collaboration Tool to evaluate the risk of bias in randomized clinical trials (RoB 2) [20, 28].

**Fig. 1:**
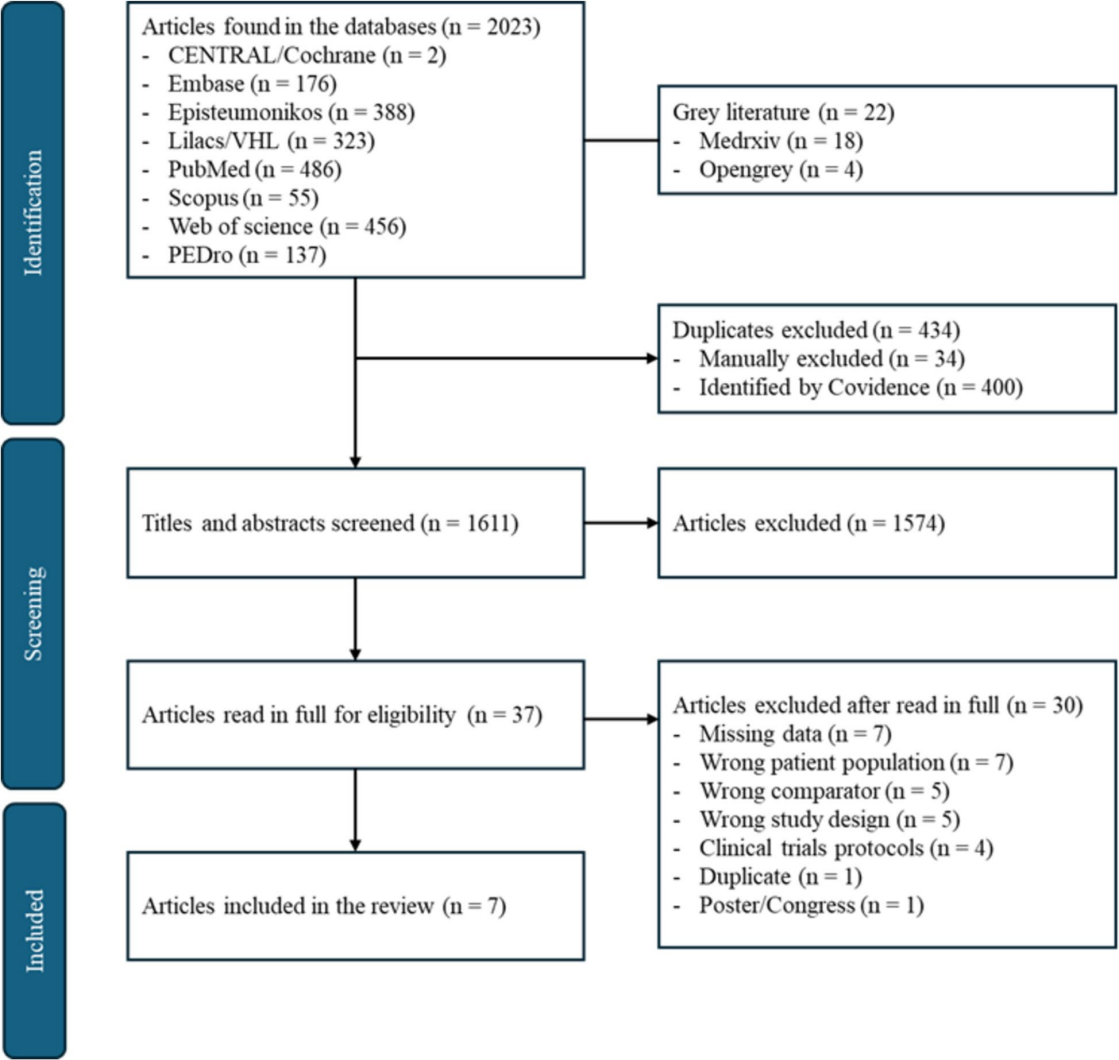
Prism flow diagram

The study selection followed the Preferred Reporting Items for Systematic Reviews and Meta-analyses (PRISMA) flowchart, as shown in Fig. 1.

#### Data extraction and management

Two reviewers (ACC and PRG) independently extracted the data using a standardized tool developed based on the Cochrane Collaboration recommendations. Any discrepancies were discussed and resolved by consensus. Disagreements in the extracted data were resolved with a third reviewer (AMI) if necessary. For this final stage, no automated tools were used; instead, discussion and consensus were achieved through a meeting.

Data collection included the general characteristics of the study (author, year, title, journal, country and language of publication, source of funding, study design, sample size); participants (age, sex, time of persistent symptoms, specific characteristics, sample size); intervention data (description of the technique, duration of the intervention, duration of sessions, attendance/frequency of participants, face-to-face or telerehabilitation, follow-up time); control data (no intervention or WHO guidelines recommendation); and data related to the outcomes (measurement methods, self-report or third-party evaluation, periods for evaluation, and follow-up time after the end of the intervention, adverse events, limitations of the study, and study conclusion).

#### Risk of bias assessment

Two reviewers independently evaluated the risk of bias using the RoB 2 tool version 2019 [28]. Differences were resolved by a third reviewer if necessary. According to Sterne et al*.* [28], the five domains for evaluating clinical trials (CTs) were (1) bias resulting from the randomization process, (2) bias due to deviations from the intended interventions, (3) bias due missing outcome data, (4) bias in the outcome measurement, and (5) bias in the selection of the reported outcome.

#### Certainty of evidence: Cochrane GRADE assessment

The evaluation of the certainty of evidence is the measurement of the level of confidence that can be placed in each estimated effect. This assessment was performed for each analyzed outcome, resulting in a classification based on the levels of evidence [29].

The factors used as a reference to raise or lower the certainty of evidence from the studies were study design, methodological limitations (risk of bias), inconsistency (heterogeneity), indirect evidence, imprecision (sample size smaller than 240–300 participants, high confidence interval), publication bias, magnitude of effect, and residual confounding factors [29]. For the indirectness domain, if there were significant differences in intervention, population, or outcome, the evidence level would be downgraded for indirectness. To assess reporting bias, a comparison was conducted on the study protocol with the published report, a search was conducted for unpublished studies in the grey literature, a comprehensive search was conducted, and industry sponsorship was checked.

If we had included more than 10 studies, we would have assessed the presence of small-study effects in paired comparisons graphically using a funnel plot.

The Grading of Recommendations Assessment, Development and Evaluation (GRADE) system was developed to grade the certainty of evidence and the strength of health recommendations [29]. According to GRADE [29], the certainty of evidence for each outcome can be classified as:

- High: strong confidence that the true effect is close to the estimated.
- Moderate: moderate confidence in the estimated effect.
- Low: limited confidence in the estimated effect.
- Very low: very limited confidence in the estimated effect with an important degree of uncertainty in the findings.

#### Data synthesis

Data were analyzed according to the Cochrane Handbook for Systematic Reviews of Interventions [20]. The scores from the immediate post-intervention results were extracted from the included studies, which were collected through continuous data (mean and standard deviation) and the total number of participants. When numerical data were missing, the authors were contacted [30–32] requesting additional data for analysis. Longobardi et al. provided the data [31]. Data meta-analysis was performed using the Review Manager analysis software, version 5.3, to quantify the results. Continuous variables were performed based on the last evaluation, and the mean difference between groups with a 95% confidence interval (CI) was calculated. Statistical significance was defined as *p* ≤ 0.05. A random-effects model was utilized for the meta-analysis to account for the variability between studies**.** There were no dichotomous variables to be analyzed. Statistical heterogeneity among the studies was evaluated using the Higgins inconsistency test (I^2^), which describes the percentage of variability in effect estimation attributed to heterogeneity. I^2^ values between 0% and 25% indicated mild, acceptable heterogeneity, 25% and 50% indicated moderate heterogeneity, and > 50% indicated high heterogeneity [28, 29].

#### Subgroup analysis and heterogeneity assessment

Post-COVID conditions affected people of different age groups and levels of acute COVID-19 infection. Therefore, differences in the population, such as age and severity of COVID-19 in the acute phase, enable relevant clinical heterogeneity. Another heterogeneity factor was using different instruments to evaluate the same outcome. The difference in the effect of the intervention can lead to statistical heterogeneity. Thus, CIs for results of individual studies (represented graphically by horizontal lines) that have little overlap likely indicate the presence of statistical heterogeneity (variability in the effects of the intervention being evaluated across the different studies) [20, 29].

#### Sensitivity analysis

To address heterogeneity in the comparison of MPT vs. control for the outcome of fatigue perception [20, 29], we conducted a sensitivity analysis excluding the study by Elhamrawy et al. [33]. The rationale for excluding this study was that the study population consisted predominantly of elderly individuals, which could significantly influence the FSS scale scores. A subgroup analysis to assess variation in treatment effects across different patient or trial subgroups was not performed due to the limited number of included studies.

## Results

Figure 1 summarizes the article selection process. A total of 2045 studies were identified through database searches. However, 434 duplicates were excluded, with 400 identified automatically by the platform and 34 manually excluded. After the screening process, seven studies were selected for data extraction.

Title and abstract screening were performed in 1611 studies; this process excluded 1574 studies, and the remaining 37 were read in full for eligibility decision. Thirty articles were excluded for the following reasons: missing data on the time of persistent symptoms [34–40], adult population restricted to a type of professional class [41], population with symptoms of fatigue and dyspnea for less than 12 weeks [42–48], RCT post-hoc [49], ongoing clinical trial protocol [50–53], congress summary [54, 55], wrong comparator [56–59], studies whose design did not correspond to RCT [60–62], and duplicate study previously excluded in the screening process [63]. The complete list of excluded articles is available in Supplementary Material 2.

### Studies characteristics

Seven RCTs were included, published between 2022 and 2023 [30–33, 64–66]. Four studies are from Spain [30, 32, 64, 66], one from Brazil [31], one from Jordan [33], and one from the UK [65]. Despite their diverse location, all selected articles were published in English.

Regarding the funding allocated for research, six studies reported receiving funding to conduct their studies [30–32, 64–66], while one study declared that it had not received any funding [33]. The sample size ranged from 26 to 148 participants. Considering the analysis to intent to treat, the total number of participants was 449, and the mean age ranged from 30 to 70 years. The female percentage varied from 35 to 99% among the studies.

The screening for ME/CFS symptoms was performed in one study [66], but no diagnosis was found in any study participant. Table 1 shows the characteristics of the included studies. The participants received IMT [30, 32, 64, 65] or MPT [30, 31, 33, 66], and one study analyzed the association between the two types [30]. In three studies, the control groups did not receive any intervention [30, 33, 65]; in another three, they followed the WHO recommendations for PCC self-management [30, 31, 66]; and in one study, they used similar placebo devices lacking the pressure valve [64]. Most studies [30, 63, 64] performed IMT for 8 weeks, with only one study using 12 weeks [32]. All studies performed IMT remotely, and the inspiratory resistance varied across studies, ranging from 25 to 80% of MIP [30, 32, 63, 64]. To evaluate the effectiveness of IMT, most studies [30, 32, 65] analyzed the effects at the end of the intervention, whereas only the study by Del Corral et al. [64] measured outcomes midway through the treatment period. Table 2 shows the details of the protocols used.

**Table 1:** Characteristics of the included studies

Author	Country	Population	Age, mean (SD)	Female sex (%)	Participants intervention (*n*)	Participants control (*n*)	Outcomes measured
Jimeno-Almazán et al. (2023) [30]	Spain	Adults hospitalized with PASC N = 84	45.3 (± 8.0)	55 (69%)	1) MPT (21) 2) IMT (17) 3) CTRM (25)	CON (21)	**PO:** Cardiorespiratory capacity (V̇O_2_max in the cycle ergometer) and Muscle strength (BP, HGS, and HSQ) **SO:** Severity of symptoms (SF-12, PCFS, mMRC, FSS, CFQ-11, GAD-7, and PHQ-9)
Longobardi et al. (2023) [31]	Brazil	Adults with PASC previous ICU hospitalization in the acute COVID-19 phase *N* = 50	60.8 (± 7.1) to 61.2 (± 7.7)	25 (50%)	MPT (25)	CON (25)	**PO:** HRQoL (SF-36), Cardiorespiratory capacity (V̇O_2_peak in the CPET modified Balke protocol), Pulmonary function (FEV_1_, FVC, FEV_1_/FVC, VE/VCO_2_, PEF, and PIF), Muscle strength (HGS, STS 30 s, TUG), Body composition (Waist and hip circumferences), Functionality (PCFS), Persistent symptoms (FSS, BAI, *BDI*), and Level of physical activity (IPAQ)
Palau et al. (2022) [32]	Spain	Adults with PASC after previous hospitalization for pneumonia COVID-19 *N* = 26	50.4 (± 12.2)	11 (42.3%)	IMT (13)	CON (13)	**PO:** Pulmonary function (VE/VCO_2_) **SO:** Cardiorespiratory capacity (V̇O_2_max and V̇O_2_peak in the CPET) and HRQoL (EQ-5D-3L)
Elhamrawy et al. (2023)[33]	Jordan	Adults with post-COVID-19 symptoms *N* = 54		19 (35%)	TC (18) MPT: (18)	CON (18)	**PO:** Muscle strength (HGS), Fatigue (FSS), Physical performance evaluation (ACT 30 s, STS 30 s), Equilibrium (TUG), and Cardiorespiratory capacity (2 min of STEP)
Del Corral et al. (2023) [64]	Spain	Adults with PASC and fatigue and dyspnea symptoms N = 88	IMT = 48.9 (8.3) IMTp = 45.3 (12.8) RMT = 45 (10.2) RMTp = 46.5 (9.6)	63 (88%)	1) IMT (22) 2) RMT (22)	3) IMTp (22) 4) RMTp (22)	**PO:** HRQoL (EQ-5D-5L and overall health by VAS), Exercise tolerance (HR–Ruffier test) **SO:** Respiratory muscle function (MIP, MEP, and IME), Pulmonary function (FVC, FEV_1_, and FEV_1_/FVC), Peripheral muscle strength (STS 1 min and HGS), Cognitive status (MoCA-S) and psychological (HADS, PTSD, PCL-C)
McNarry et al. (2022) [65]	United Kingdom	Adults recovering from autocorrelated COVID-19 *N* = 148	46.6 (± 12.2)	(88%)	IMT (111)	CON (37)	**PO:** HRQoL (K-BILD); **SO:** Dyspnea (BDI- TDI), IMS (MIP, SMIP, and FIT), Cardiorespiratory capacity (V̇O_2_max no Chester Step Test), Daily living activities (GT9X accelerometer with non-dominant wrist) and Mental health and well-being (Treatment Self-Regulation Questionnaire-15 question and Perceived competence scale)
Jimeno-Almazán et al. (2022) [66]	Spain	Adults with PASC after mild acute infection of COVID-19 *N* = 39	45.2 (± 9.5)	29 (74.35%)	MPT (13)	CON (16)	**PO:** Severity of symptoms (SF-12, GAD-7, PHQ-9, mMRC, CQF-11, FSS, and PCFS), Cardiorespiratory capacity (FEV_1_, FVC, FEV_1_/FVC Physical condition (V̇O_2_max in the Ekblom-Bak protocol and Muscle strength (HGS, 5-STS, BP, HSQ, 3 s isometric knee extension test at 110° knee flexion)

*ACT* Arm Curl Test, *BAI* Beck Anxiety Inventory, *BDI* Baseline Dyspnea Index, *BDI* Beck Depression Inventory, *BP* Bench Press, *CFQ*-*11* Chalder Fatigue Scale, *CON* Control, *CPET* Cardiopulmonary Exercise Testing, *CTRM* Concurrent Training Program [with inspiratory muscle training], *EQ*-*5D*-*3L* EuroQol-3D questionnaire, *EQ*-*5D*-*5L* EuroQol-5D questionnaire, *FEV*_*1*_ Forced Expiratory Volume in one second, *FIT* Fatigue Index Test, *FSS* FatigueSeverity Scale, *FVC* Forced Vital Capacity, *GAD*−7 General Anxiety Disorder Questionnaire-7, *HADS* Hospital Anxiety and Depression Scale, *HGS* Handgrip Strength, *HR* Heart Rate, *HRQoL* Health-related Quality ofLlife, *HSQ* Half Squat, *IME* Inspiratory Muscle Endurance, *IMS* Inspiratory Muscle Strength, *IMT* Inspiratory Muscle Training, *IMTp* Inspiratory Muscle Training placebo, *IPAQ* International Physical Activity Questionnaire, *K*-*BILD* King’s Brief Interstitial Lung Disease, *MEP* Maximal Expiratory Pressure, *MIP* Maximal Inspiratory Pressure, *mMRC* Modified Medical Research Council Dyspnea Scale, *MoCA*-*S* Montreal Cognitive Assessment, *MPT* Multicomponent Physical Training, *MPTp* Multicomponent Physical Training placebo, *PCFS* Post-COVID-19 Functional status Scale, *PCL*-*C* Post-traumatic stress disorder Checklist-Civilian version, *PEF* Peak Expiratory Flow, *PHQ*-*9* Patient Health Questionnaire-9, *PIF* Peak Inspiratory Flow, *PO* Primary Outcome, *PTSD* Post-Traumatic Stress Disorder, *RMT *Repiratory Muscle Training, *RMTp* Repiratory Muscle Training placebo, *SD* Standard Deviation, *SF*-*12* The 12-Item Short-Form Health Survey, *SMIP* Sustained Maximal Inspiratory Pressure, *SO* Secondary Outcome, *STS* Sit-To-Stand, 5-*STS* 5 times Sit-To-Stand, *TC* Tai Chi, *TDI* Transition Dyspnea Index, *VAS* Visual Analog Scale, *VCO*_*2*_ Carbon Dioxide Production, *VE* minute ventilation, *V̇O*_*2max*_ maximal volume of oxygen, *V̇O*_*2*_*peak* peak consumption of O_2_, *TUG* Time Up and Go

**Table 2:** Details of the inspiratory muscle training protocols of the included studies

	**Jimeno-Almazán et al. (2023) **[30]	**Palau et al. (2022) **[32]	**Del Corral et al. (2023) **[64]	**McNarry et al. (2022) **[65]
Protocol	Warm-up: 1 set of 3 repetitions to determine MIP; Training: 1 set of 30 repetitions in a 62.5% (± 4.6%) MIP	Warm-up: 1 set to determine the MIP; Training: 25–30% of MIP. The resistance was modified in each session accordingly, with 25% to 30% of weekly MIP measured	Warm-up: 3 min of breaths in a 20% MIP and 1 min rest; Training: 10 repetitions of 6 breathing cycles of 1 min and 30 s in a 50–80% MIP with resting between the cycles of 1 min	Warm-up: 1 max inspiratory from RV to determine 80% SMIP; Training: perform maximal inspiratory > 80% of MIP until inspiratory failure. Each session involved up to six blocks of six inspirations, with the rest periods interspersing each inspiration progressively decreasing from 40 to 10 s with each block
Frequency (day/week)	2 sessions/day; 7 days/week	2 sessions/day; weekly frequency not informed	2 sessions/day; 6 days/week	2 sessions/day; nonconsecutive days of the week
Duration of each session	Not informed	20 min	2 min	Max 20 min
Supervision	No	Semi-supervised	Supervised in the night sessions	No
Follow-up	8 weeks	12 weeks	8 weeks	8 weeks
Control	WHO recommendations	No treatment	A device similar to Threshold, without a resistance valve	Waiting list for usual care

*WHO* World Health Organization, *MIP* Maximal Inspiratory Pressure, *SMIP* Sustained Maximal Iinspiratory Pressure, *IMT* Inspiratory Muscle Training, *RV *Residual Volume

### Risk of bias assessment

The overall risk of bias in each article is summarized in Table 3. Of the seven included studies, two were assessed as having a high risk of bias [30, 66], two as “some concern” [33, 65], while three were classified as having a low risk of bias [31, 32, 64]. In two of the seven included studies, domain 2–deviation from intended intervention was evaluated as “some concern.” Regarding domain 2, it is important to highlight that studies involving exercise as an intervention are more challenging to blind both the patient and the intervention team. The studies by Elhamrawy et al. [33], Jimeno-Almazán et al. [66], and Jimeno-Almazán et al. [30] did not specify whether participant assessments were conducted in a blinded manner. Regarding the selection of reported outcomes, the studies by Jimeno-Almazán et al. [66] and Jimeno-Almazán et al. [30] evaluated the same outcome (fatigue) using two different scales, representing another factor contributing to an increased risk of bias and, consequently, a lower certainty of evidence.

**Table 3:**
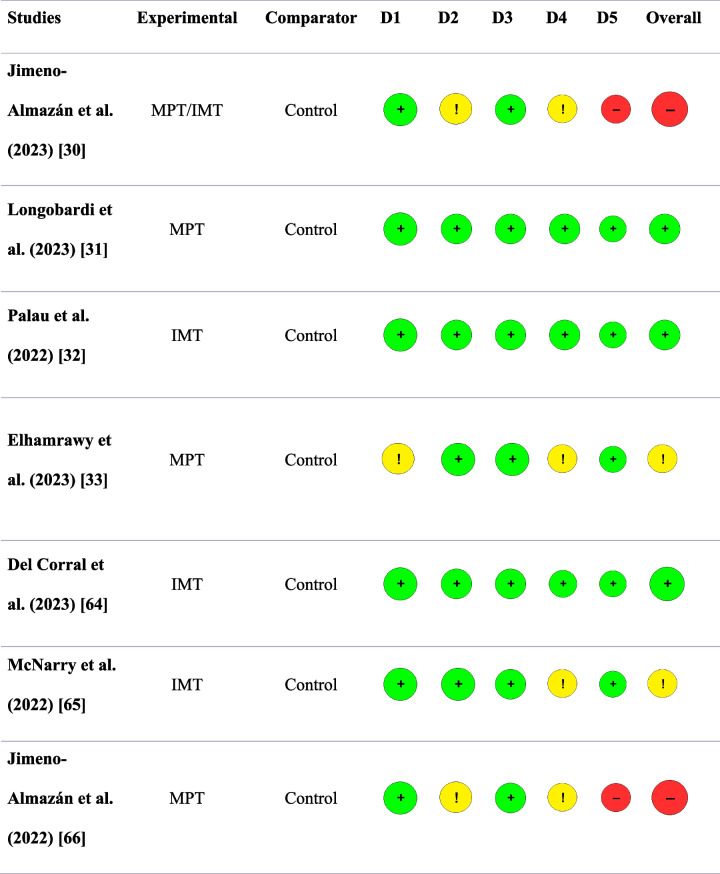
Overall results of risk of bias of each study [30–33, 64–66]

*IMT* inspiratory muscle training, *MPT* multicomponent physical training, *D1* randomization process, *D2* deviations from intended interventions, *D3* missing outcome data, *D4* measurement of results, *D5* selection of reported results; Overall

### Effects of inspiratory muscle training vs. no intervention/WHO recommendations

#### Pulmonary function

**Fig. 2:**
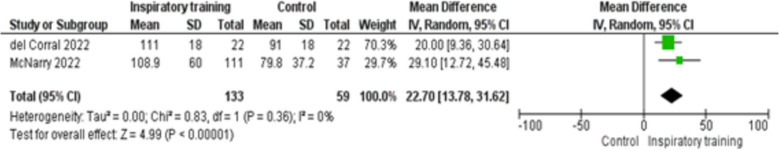
Forest plot of the effects of IMT on MIP

The MIP parameter was used to evaluate respiratory muscle strength. The meta-analysis, based on data from two studies [64, 65], demonstrated a statistically significant improvement in respiratory muscle strength with IMT (MD = 22.70; 95% CI: 13.78 to 31.62; *p* < 0.00001; I^2^ = 0%) (Fig. 2). The effect is clinically relevant, as Del Corral et al. [67] reported that a variation of 18 cm H_2_O is clinically significant. No statistical heterogeneity was observed between studies.

The parameters forced vital capacity (FVC), forced expiratory volume in 1 s (FEV_1_), and FEV_1_/FVC were analyzed only by the study by Del Corral et al. [64], in which only FVC showed a statistically significant improvement with IMT (MD = − 10; 95% CI: − 19.53 to − 0.47). No statistical difference were observed in FEV_1_ (MD = 9; 95% CI: − 0.76 to 18.76; *p* = 0.07) or FEV_1_/FVC (MD = 0; 95% CI: − 3.01 to 3.01; *p* = 1) with IMT. It is important to highlight that no minimal clinically important difference (MCID) has been established for FEV_1_, FVC, and FEV_1_/FVC, as these are fixed values used to determine the presence and severity of pulmonary function impairment.

#### Cardiorespiratory capacity

**Fig. 3:**
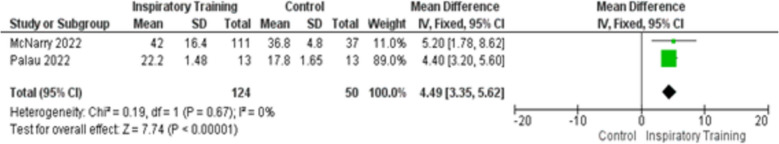
Forest plot of the effects of IMT on $\dot{V}$ O_2max_

One study used the Ruffier test [63], and three studies [30, 32, 65] evaluated maximal volume of oxygen ($\dot{V}$ O_2max_); however, only two [30, 65] provided sufficient data for meta-analysis. A statistically significant improvement in $\dot{V}$ O_2max_ was observed in the IMT group (MD = 4.49; 95% CI: 3.35 to 5.62; *p* < 0.00001; I^2^ = 0%) (Fig. 3). The result is clinically significant, as according to Lang et al. [68], for every 1 MET (3.5 ml O₂/kg/min) increase, mortality decreases by 12%.

Del Corral et al. [64] found no significant changes in the Ruffier index with the IMT. Jimeno-Almazán et al. [30] did not provide the data for our meta-analysis and did not find statistically significant differences in $\dot{V}$ O_2max_ between the groups after IMT.

#### Upper limb muscle strength

Two studies [30, 64] evaluated handgrip muscle strength (HGS), but data for meta-analysis were available only in one study (MD = − 0.6; 95% CI: − 6.07 to 4.87, *p* = 0.83) [64]. No statistically significant increase in HGS was observed after IMT, and no clinical improvement was observed, considering that the MCID for the HGS test ranges from 5.0 to 6.5 kg [69].

Jimeno-Almazán et al. [30] also performed the progressive load test of the percentage of 1MR of the straight bench press, with no clinical and statistical improvement.

#### Lower limb muscle strength

Lower limb muscle strength was evaluated using the timed sit-to-stand (STS) test [64] and 1MR half squat percentage progressive load tests [30]. A clinically and statistically significant improvement in the timed STS test was observed with IMT [64] (MD = 7.40; 95% CI: 2.43 to 12.37, *p* = 0.03), considering that a difference of ≥ 2 repetitions represented the MCID as reported by Zanini et al. [70]. Regarding the 1MR half squat load test, no improvement was found in the progressive 1MR half squat load test [30]. Due to unavailable data, an estimated effect could not be calculated for Jimeno-Almazán et al. [30].

#### Perception of fatigue

Two studies [30, 64] evaluated this outcome. Based on the narrative synthesis, Del Corral et al. [64] found no significant reduction in the number of reports of fatigue with IMT.

Jimeno-Almazán et al. [30] was the only study to analyze this outcome through fatigue perception scales, with no significant difference between groups after IMT.

#### Physical functioning

**Fig. 4:**
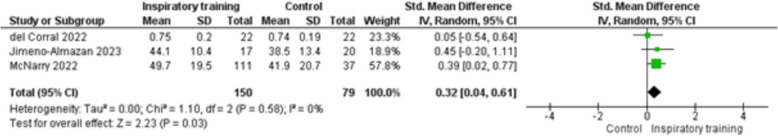
Forest plot of the effects of IMT on physical functioning

Based on the narrative synthesis, physical functioning was not different between the control and IMT groups in the study that evaluated it by Post-COVID-19 Functional Status Scale (PCFS) [30]. Four studies evaluated physical functioning [30, 32, 64, 65] and three provided data for meta-analysis [30, 64, 65] (Fig. 4). There was a statistically significant improvement in physical functioning; however, based on Higgins et al. [20], the effect is considered small (SMD = 0.32; 95% CI: 0.04 to 0.61; *p* = 0.03; I^2^ = 0%).

The results of Palau et al. (2022) [32] were available in graphical format. According to the author, there was a significant improvement in the IMT group (− 0.31, 95% CI − 0.54 to − 0.07, *p* = 0.013).

#### Perception of dyspnea

Dyspnea was evaluated in three studies [30, 64, 65] using dyspnea complaints [64], the Modified Medical Research Council Dyspnea (mMRC) scale [30], and the Baseline Dyspnea Index (BDI) and Transition Dyspnea Index (TDI) [65]. Del Corral et al. [64] reported a significant decrease in dyspnea complaints, and McNarry et al. [65] likewise observed a greater decrease in dyspnea with IMT. Jimeno-Almazán et al. [30] reported no difference between groups. Meta-analysis was not performed due to the insufficient available data.

#### Treatment adherence

The minimum adherence required in the studies ranged from 67% [32] to 85% [30, 32, 64]. Treatment adherence in the IMT groups was high in most studies, ranging from 95% [64] to 100% [30, 32] of patients, according to information from the follow-up records. In the study by McNarry et al. [65], a high dropout rate of 69% was observed, with the highest dropout in the intervention group; only 31% of the participants completed the study and were included in the protocol analysis.

#### Adverse events

No adverse effects were reported during or after IMT [30, 32, 64]. In the study by Del Corral et al. [64], one patient in the control group experienced a worsening of symptoms, indicating that the worsening was not associated with the intervention. McNarry et al. [65] did not evaluate adverse events in their study.

#### Certainty of evidence

The certainty of evidence was considered low for all outcomes (MIP, $\dot{V}$ O_2max_, and physical functioning). This is presented in the summary of evidence (Table 4).

**Table 4:** Summary of evidence of inspiratory muscle training vs. Control

		**Certainty assessment**					**Summary of results**				
**Participants** **(studies)** **Follow-up**	**Risk of bias**	**Inconsistency**	**Indirect evidence**	**Imprecision**	**Publication bias**	**Overall certainty of evidence**	**Study event rates (%)**		**Relative effect (95% CI)**	**Potential absolute effects**	
							**Control**	**Inspiratory exercise**		**Risk with control**	**Risk difference with inspiratory exercise**
MIP (cmH_2_O) (%pred)											
192 (2 RCTs)	High^a^	Moderate	Moderate	High^b^	None	⨁⨁◯◯ Low	59	133	–	59	MD 22.7 higher (13.78 higher to 31.62 higher)
Physical functioning											
229 (3 RCTs)	High^c^	Moderate	Moderate	High^b^	None	⨁⨁◯◯ Low	79	150	–	–	SMD 0.32 higher (0.04 higher to 0.61 higher)
VO_2_ ml/kg/min											
174 (2 RCTs)	High^a^	Moderate	Moderate	High^b^	None	⨁⨁◯◯ Low	50	124	–	50	MD 4.49 higher (3.35 higher to 5.62 higher)

*95*% *CI* 95% confidence interval, *MD* mean difference, *MIP* maximal inspiratory pressure, *SMD* standardized mean difference, *VO*_*2*_ maximal volume of oxygen

^a^McNarry et al. (2022) [65] showed high risk of bias

^b^Sample less than 400 participants

^c^McNarry et al. (2022) [65] and Jimeno-Almazan et al. (2023) [30] showed high risk of bias

### Effects of multicomponent physical training vs. no intervention/WHO recommendations

Multicomponent physical training was analyzed in four studies [30, 31, 33, 66], and all evaluated the effects shortly after the intervention ended. The duration of MPT ranged from 8 to 16 weeks, with a mean training period of 11 weeks.

The weekly frequency of MPT ranged from three [30, 31, 66] to four [33] sessions weekly, with sessions of 60 min in all studies [30, 31, 33, 66]. Two studies created the MPT program based on adaptations of the American College of Sports Medicine (ACSM) guideline for chronic obstructive pulmonary disease and cardiovascular diseases, while the other two did not report whether the training program was adapted from any pre-existing one [31, 33]. In three clinical trials, exercise intensity was progressively individualized according to patient tolerance [30, 31, 66], whereas one study did not specify whether load progression was implemented during the intervention [33]. The MPT summary is described in Table 5.

**Table 5:** Description of the multicomponent physical training protocols

	**Elhamrawy et al. (2023) **[33]	**Jimeno-Almazán et al. (2023) **[30]** Jimeno-Almazán et al. (2022)**[66]	**Longobardi et al. (2023) **[31]
Exercises intensity	40–60% of HRmax or Borg scale 4–6; fixed weigh load of 0.5 kg for strengthening	2 days resistance training (1MR 50%), combined with varied training of moderate intensity (HRmax varying 55–70–80%), and 1 light training day (HRmax varying 65–70%)	Based on the number of repetitions/series, difficulty level of execution, and duration of the exercise
Details of the exercises performed	Static stretching of the trunk and limbs (10 min); Strengthening of upper and lower limbs (20 min); Aerobic exercise (15–20 min of moderate walking on a treadmill); Warm-up (10 min of stretching)	Resistance training (3 sets, 8 reps of squats, bench press, deadlift, and bench press), combined with moderate intensity training (4–6 reps in 3–5 min of the same exercises): Continuous light intensity training (30–60 min, HRmax 65–70%)	Aerobic exercise (10–50 min); Strengthening of trunk, upper and lower limbs (± 6 exercises, 3–5 sets of 8–15 repetitions); Static stretching of trunk and limbs (8–10 min)
MPT Frequency	4 sessions/week	3 sessions/week	3 sessions/week
Duration of each session	60 min	60 min	60 min
Supervision	Supervised in the night sessions	Supervised	Semi-supervised
Progression of intensity	Not informed	Modified Borg scale and PCFS	Modified Borg scale and PCFS
Follow-up	12 weeks	8 weeks	16 weeks
Control	No treatment	WHO recommendations	No treatment

#### Pulmonary function

**Fig. 5:**
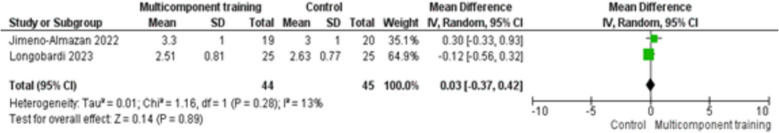
Forest plot of the effects of MPT on FEV_1_

**Fig. 6:**
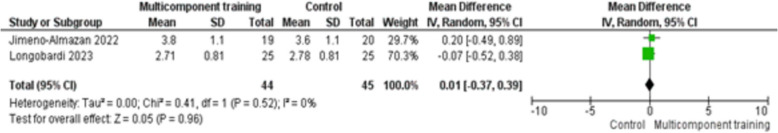
Forest plot of the effects of MPT on FVC

**Fig. 7:**
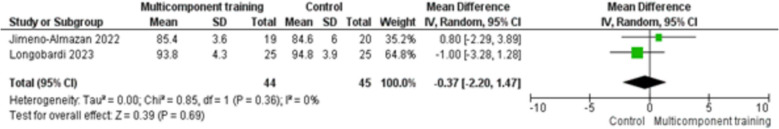
Forest plot of the effects of MPT on FEV_1_

Two studies [31, 66] evaluated pulmonary function using FEV_1_, FVC, and FEV_1_/FVC parameters. The MPT group showed no statistically significant improvement in FEV_1_ (MD = 0.03; 95% CI: − 0.37 to 0.42; p = 0.89; I^2^ = 13%) (Fig. 5), FVC (MD = 0.01; 95% CI: − 0.37 to 0.39; *p* = 0.96; I^2^ = 0%) (Fig. 6) or FEV_1_/FVC ratio (MD = − 0.37; 95% CI: − 2.20 to 1.47; *p* = 0.39; I^2^ = 0%) (Fig. 7). It is important to highlight that no MCID has been established for FEV₁, FVC, or FEV₁/FVC, as these are fixed values used to determine the presence and severity of pulmonary function impairment.

#### Cardiopulmonary capacity

**Fig. 8:**
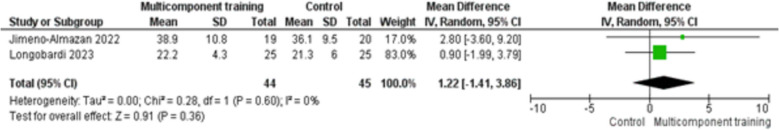
Forest plot of the effects of MPT on the $\dot{V}$ O_2max_

Three studies analyzed the effect of MPT on $\dot{V}$ O_2max_ [30, 31, 66]. Only two studies [31, 66] provided data on MPT effect to perform the meta-analysis (Fig. 8). No statistically significant change in $\dot{V}$ O_2max_ was observed with MPT (MD = 1.22; 95% CI: − 1.41 to 3.86; *p* = 0.36; I^2^ = 0%). In the study by Jimeno-Almazán et al. [30], no significant differences in $\dot{V}$ O_2max_ were found between groups.

#### Upper body muscle strength

Fatigue in upper body muscle strength was measured using the HGS test [30, 31, 33, 66], the 1MR percentage test of straight bench press [30, 66], and elbow flexion [33].

**Fig. 9:**
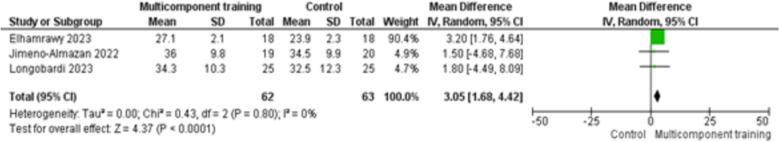
Forest plot of the effects of MPT on the upper body strength (HGS)

Three studies [31, 33, 66] provided the HGS data, showing a statistically significant improvement with MPT (MD = 3.05; 95% CI: 1.68 to 4.42; *p* < 0.0001; I^2^ = 0%) (Fig. 9). The effect has no clinical significance since changes from 5 to 6.5 kg were considered significant [69].

The HGS did not show significant improvement with MPT. In the study by Jimeno-Almazán et al*.* [30], the data from this study were not included in the meta-analysis as they were presented only in graphical form. The 1MR percentage test of the straight bench press was used in two studies [30, 66], with the MPT groups showing a significant difference in its execution. The study by Jimeno-Almazán et al. [66] showed no significant difference in the estimated effect (MD = 1.03; 95% CI: 0.36 to 1.70; *p* = 0.003; I^2^ = 0%). Elhamaray et al. [33] showed an increased number of arm flexion repetitions in 30 s.

#### Lower limb muscle strength

Fatigue in lower body muscle strength was measured using the timed STS [31, 33], Time Up and Go (TUG) [31, 33], the 1MR half squat percentage 5 times sit-to-stand (5-STS) [66], and isometric knee extension [66].

**Fig. 10:**
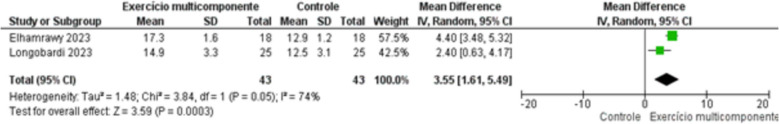
Forest plot of MPT effects on STS in 30 s

The 30-s STS test results were reported in two studies [31, 33] (Fig. 10). A statistically significant improvement was observed in the MPT group (MD = 3.55; 95% CI: 1.61 to 5.49; *p* = 0.0003; I^2^ = 74%). The effect was clinically significant since, according to Zanini et al. [70], the difference of two repetitions is considered clinically significant when the upper limit of the confidence interval is observed. However, heterogeneity between studies was high.

**Fig. 11:**
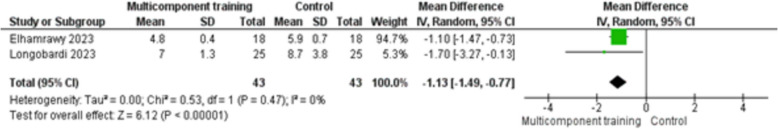
Forest plot of the effects of MPT on TUG

The TUG test, performed in two studies [31, 33], showed a statistically significant improvement in the MPT group (MD = − 1.13; 95% CI: − 1.49 to − 0.77; *p* < 0.00001; I^2^ = 0%) (Fig. 11). Despite the statistically significant difference, according to Gautschi et al*.* (2017) [71], the minimum clinical difference for TUG is 3.4 s.

The 1MR half squat percentage test was used in two studies [30, 66], with statistically significant improvements in the MMT group. However, data extraction was available only in the study by Jimeno-Almazán et al. [66] (MD = 0.93; 95% CI: 0.25 to − 1.60; *p* = 0.007). Jimeno-Almazán et al. [66] also performed the 5-STS test and the isometric knee extension test to evaluate the lower limb muscle strength. A significant improvement was observed in the 5-STS test (MD = − 150; 95%; CI: − 2.35 to − 0.65; *p* = 0.0005), but no significant change was found for the isometric knee extension test at 110° (MD = 63.70; 95%; CI: − 39.24 to − 166.64; *p* = 0.23).

#### Perception of fatigue

**Fig. 12:**
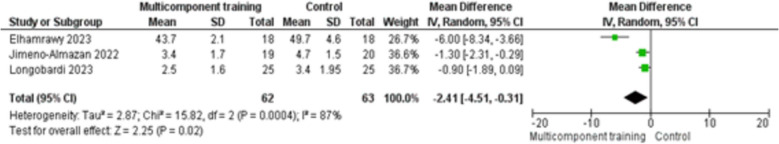
Forest plot of the effects of MPT on FSS

Self-reported fatigue symptoms were evaluated through the FSS test and Chalder fatigue scale (CFQ-11) [30, 31, 33, 66], with FSS data available only in three [31, 33, 66]. Considering that a difference of at least 0.45 points on the FSS represents a clinically significant difference in fatigue [72], a clinically and statistically significant reduction was observed in the MPT group (MD = − 2.41; 95% CI: − 4.51 to − 0.31; *p* = 0.002; I^2^ = 87) (Fig. 12). To address the high heterogeneity (87%), a sensitivity analysis was conducted by removing the study by Elhamrawy et al. (2023) [33]. This reduced heterogeneity to 0%, and the resulting effect estimate, based on two studies [31, 66], continued to show a statistically significant reduction in fatigue in favor of MPT (MD = − 1.10; 95% CI: − 1.80 to − 0.39).

In the study by Jimeno-Almazán et al. [30], FSS values improved significantly in the MPT group. Using the CFQ-11, a reduction in perceived fatigue was also reported in two studies [30, 66].

#### Physical functioning

**Fig. 13:**
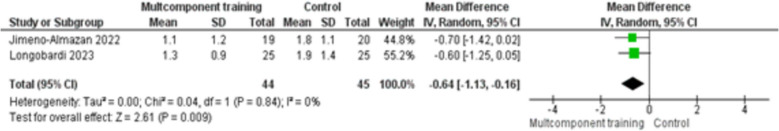
Forest plot of the effects of MPT on PCFS

**Fig. 14:**
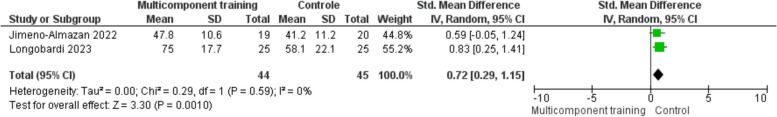
Forest plot of the effects of MPT on physical functioning

Three studies [30, 31, 66] evaluated the participants using the PCFS scale; however, data were available in two [30, 66]. A statistically significant improvement in PCFS was observed between groups, expressed by a reduction in total PCFS score (MD = − 0.64; 95% CI: − 1.13 to − 0.16; *p* = 0.009), as shown in Fig. 13. No heterogeneity was detected between studies. Jimeno-Almazán et al. [30] also reported significant improvement in the MPT group. Although no MCID for the PCFS scale was identified in the literature, it is relevant to highlight that the MPT group showed a score of approximately 1, compared to around 2 in the control group. The PCFS scale classifies 1 as negligible limitations and 2 as slight limitations. Based on the physical component of the 12-Item Short-Form Health Survey (SF-12) questionnaire [65] and the 36-Item Short-Form Health Survey (SF-36) [31], a large effect [20] and statistically significant improvement were observed in physical functioning in the MPT group (SMD = 0.72; 95% CI: 0.29 to 1.15; *p* < 0.001; I^2^ = 0%) (Fig. 14).

#### Perception of dyspnea

Two studies [30, 66] evaluated this outcome using the mMRC scale. The meta-analysis was not performed because only one study [65] presented the data. Jimeno-Almazán et al. [66] found no significant difference in the perception of dyspnea between groups (MD = − 1.24; 95% CI: − 1.24 to 0.05; *p* = 0.07). According to Jimeno et al. [30], the number of participants with mMRC < 2 increased from 55 to 79%, indicating a statistically significant reduction in the perception of dyspnea after MPT. It is important to note that the mMRC scale ranges from 0 to 4, with 4 representing the most severe degree of dyspnea [73].

#### Treatment adherence

Two studies determined a minimum frequency of 85% [30, 66]. In the study by Jimeno-Almazán et al. [66], one participant in the MPT group abandoned the program due to low adherence. In the study by Jimeno-Almazán et al. [30], three patients withdrew the study for reasons unrelated to the worsening of symptoms: two were in the IMT with MPT combined group, one had moderate SARS-CoV-2 reinfection, and the other due to instability of a psychiatric pathology, and one was in the MPT group and withdrew due to non-adherence to training. Longobardi et al. [31] reported the loss of nine participants during the treatment period, four in the MPT group and five in the control group; none were related to the study or the training protocol.

Three studies [30, 31, 66] reported adherence rates to MPT among patients who completed the study, ranging from 71.2 to 88%. Elhamrawy et al. [33] did not provide data on treatment adherence.

#### Adverse events

No adverse effects were reported during or after MPT [30, 31, 66]. Elhamrawy et al. [33] did not mention the adverse events in their study.

#### Certainty of evidence

The certainty of evidence was rated as low for all outcomes (FEV₁, FVC, FEV₁/FVC, V̇O₂max, HGS, STS, TUG, PCFS, and physical functioning), as the risk of bias across studies ranged from low to high. These findings are summarized in the overall risk of bias assessment (Table 3) and the summary of evidence (Table 6).

**Table 6:** Summary of evidence of multicomponent physical training

		**Certainty assessment**					**Summary of results**				
**Participants** **(studies)** **Follow-up**	**Risk of bias**	**Inconsistency**	**Indirect evidence**	**Imprecision**	**Publication bias**	**Overall certainty of evidence**	**Study event rates** **(%)**		**Relative effect (95% CI)**	**Potential absolute effects**	
							**Control**	**Multicomponent training**		**Risk with control**	**Risk difference with multicomponent training**
Physical functioning											
129 (3 RCTs)	High^a^	Moderate	Moderate	High^b^	None	⨁⨁◯◯ Low	65	64	–	–	SMD 0.75 higher (0.39 higher to 1.1 higher)
FSS											
125 (3 RCTs)	High^a^	Moderate^c^	Moderate	High^b^	None	⨁⨁◯◯ Low	63	62	–	63	MD 1.51 lower (2.18 lower to 0.83 lower)
HGS											
125 (3 RCTs)	High^a^	Moderate	Moderate	High^b^	None	⨁⨁◯◯ Low	63	62	–	63	MD 3.05 higher (1.68 higher to 4.42 higher)
STS											
86 (2 RCTs)	Moderate	High^d^	Moderate	High^b^	None	⨁⨁◯◯ Low	43	43	–	43	MD 3.55 higher (1.61 higher to 5.49 higher)
TUG											
86 (2 RCTs)	High	Moderate	Mpderate	High^b^	None	⨁⨁◯◯ Low	43	43	–	43	MD 1.13 lower (1.49 lower to 0.77 lower)
FEV_1_(L) (%pred)											
89 (2 RCTs)	High^a^	Moderate	Moderate	High^b^	None	⨁⨁◯◯ Low	45	44	–	45	MD 0.03 higher (0.37 lower to 0.42 higher)
FVC(L) (%pred)											
89 (2 RCTs)	High^a^	Moderate	Moderate	High^b^	None	⨁⨁◯◯ Low	45	44	–	45	MD 0.01 higher (0.37 lower to 0.39 higher)
FEV_1_/FVC%											
89 (2 RCTs)	High^a^	Moderate	Moderate	High^b^	None	⨁⨁◯◯ Low	45	44	–	45	MD 0.37 lower (2.2 lower to 1.47 higher)
PCFS											
89 (2 RCTs)	High^a^	Moderate	Moderate	High^b^	None	⨁⨁◯◯ Low	45	44	–	45	MD 0.64 lower (1.13 lower to 0.16 lower)
$\dot{V}$O_2_ max ml/kg/min											
89 (2 RCTs)	High	Moderate	Moderate	High^b^	None	⨁⨁◯◯ Low	45	44	–	45	MD 1.22 higher (1.41 lower to 3.86 higher)

*95*% *CI* 95% confidence interval, *MD* mean difference, *MIP* maximal inspiratory pressure, *SMD* standardized mean difference, *FEV*_*1*_ forced expiratory volume in one second, *FVC* forced vital capacity, *HGS* handgrip strength, *FSS* fatigue severity scale, *PCFS* post-COVID-19 functional status scale, *STS* sit-to-stand, *TUG* time up and go, *VO*_*2*_*max* maximal volume of oxygen

^a^Risk of bias varied from some concern to high

^b^Sample less than 400 participants

^c^I² = 87%. There was variability between the age of the participants (elderly, adults) and the severity of COVID-19

^d^I² = 70–100%. There was variability between the age of the participants (elderly, adults) and the severity of COVID-19

## Discussion

The objective of this systematic review was to evaluate the effectiveness of IMT and MPT in reducing signs and symptoms of fatigue and dyspnea in individuals with PCC. Physical functioning, treatment adherence, and adverse events were also assessed as secondary factors.

It was expected that IMT would improve dyspnea, as occurs in patients with Interstitial lung disease (ILD) and chronic obstructive pulmonary disease (COPD), due to the reduction in mechanical pulmonary overload and increase in neural respiratory drive [74]. However, the results of this review indicate that the benefits of IMT for dyspnea remain uncertain, based on findings from the three RCTs that evaluated this outcome [30, 64, 65]. Two studies [64, 65] reported a reduction in dyspnea, while one [30] found no significant difference. Despite the pulmonary origin of COVID-19 and its potential impact on respiratory muscles, current evidence is insufficient to conclude that strengthening inspiratory muscles reduces dyspnea associated with PCC.

The IMT showed statistically significant improvement in MIP, $\dot{V}$ O_2max_, and physical functioning, consistent with the findings of a similar systematic review evaluating the same intervention in patients with PCC [75]. However, the improvements in $\dot{V}$ O_2max_ did not provide significant changes in physical functioning and peripheral muscle strength or reduce the perception of dyspnea and fatigue in individuals with PCC, contrasting the evidence of improvement demonstrated in patients with pulmonary pathologies [76–78]. Although these conditions share chronic systemic functional consequences, such discrepancies suggest that improving physical functioning in PCC may depend to an even greater extent on extrapulmonary factors than is generally recognized in chronic respiratory diseases.

The discrepancy in results regarding physical functioning may be associated with the type of test used to measure this outcome. In the RCTs included in this meta-analysis, the 30-s STS was employed, whereas the improvement reported by Xavier et al. (2024) [75] was based on a narrative synthesis of the 6-min walk test.

Meta-analyses of the effects of MPT showed no significant changes in pulmonary parameters (FEV_1_, FVC, and FEV_1_/FVC) and no significant improvement in exercise tolerance as measured by $\dot{V}$O_2max_. Systematic reviews evaluating the effects of pulmonary rehabilitation or combined modalities in individuals with persistent symptoms for less than three months reported improvements in $\dot{V}$O_2max_ as a direct measurement of exercise testing [79, 80] or through indirect estimation by the 6-min walk test [79–81]. A possible explanation for the lack of significant changes in $\dot{V}$O_2max_ in our meta-analysis may be associated with a longer duration of symptoms(more than 3 months). The MPT promoted clinically relevant changes in muscle strength (HGS, STS, TUG), physical functioning, and perception of fatigue, confirming the findings of Oliveira et al. (2024) [80].

It is important to note that the score required to determine a clinically meaningful improvement in fatigue using self-reported scales remains unknown [82]. Therefore, fatigue in individuals with PCC is likely not solely associated with cardiopulmonary factors but rather involves multisystem mechanisms that are not yet fully understood, including immunological and inflammatory components [83]. Consequently, isolating the benefits of MPT to a single physiological system remains challenging. Our findings, therefore, support recent hypotheses suggesting that improvements in PCC symptoms, especially fatigue, may be best achieved through the combination of different training modalities.

No adverse events were reported with either IMT or MPT. Participant adherence was high for both trainings, supporting previous findings that IMT [84] and MPT [35] are safe and well-tolerated in individuals with PCC-related dyspnea and fatigue, thereby encouraging further research in this population.

A higher proportion of participants in the included studies were female, consistent with previous evidence indicating a higher risk of PCC symptoms in women [7, 85, 86], including persistent dyspnea and fatigue across different phases of disease severity. Several systematic reviews have examined the effectiveness of various exercise-based rehabilitation interventions for PCC [87–91]. Most of these systematic reviews included RCTs conducted during the acute phase of COVID-19 [87–90]. Reviews that exclusively analyzed RCTs [75, 79–81, 92–95] adopted a temporal criterion for symptom persistence different from our systematic review, which represents one of its main distinguishing features. Until 2022, a minimum duration of 4 weeks was considered sufficient to diagnose persistent post-COVID-19 symptoms as PCC. However, by the end of 2022, this temporal criterion was extended to 12 weeks, as recommended by the main international health organizations [11, 12, 96]. This temporal definition is relevant, as the physiological response to exercise-based rehabilitation interventions stimuli may differ between the post-acute and chronic phases and the effects of interventions may be more challenging to identify and document in individuals with long-standing symptoms compared to those recovering from acute conditions [97]. Therefore, it is important to acknowledge the methodological differences that enrich the discussion on the actual benefits of exercise-based rehabilitation interventions in PCC.

Considering the methodological differences of previous systematic reviews [87–91] published on exercise-based rehabilitation interventions for PCC, it was found that some of them [87–89] included fewer studies, others [88, 91] included studies such as non-randomized controlled trials (quasi-experimental studies), case reports, cross-sectional studies, and observational studies, or did not use GRADE to assess the certainty of evidence [87–89].

Regarding the impact of risk of bias in the included studies, it is well established that a higher risk of bias reduces the certainty of evidence. In most RCTs [30–33, 65, 66], the absence of participant blinding and the potential influence of placebo effects on perceived benefits in intervention groups represent methodological limitations that should be addressed in future research. It is also important to note that blinding is inherently more challenging in studies involving exercise interventions, both for participants and for the teams administering the intervention.

One limitation of our systematic review was the meta-analysis conducted with only two studies [31, 33], which resulted in high heterogeneity. To address this, a sensitivity analysis was performed, reducing heterogeneity to 0%, as previously detailed in the results section. The initial high heterogeneity may be attributed to variations in post-COVID diagnosis time, disease severity, and intervention duration among studies. However, we consider these differences insufficient to preclude the meta-analysis, as both studies implemented protocols involving stretching, aerobic exercise, and strengthening, thereby maintaining homogeneity.

It is important to note that variability in intervention characteristics, such as duration, frequency, and exercise intensity, is common in physiotherapy RCTs [98]. Given this heterogeneity, we employed a random-effects model to estimate the effect, as it accounts for variability between studies arising from population differences or other study-specific factors. However, it is important to highlight that the heterogeneity of these protocols makes it challenging to determine which type of exercise is most effective in improving outcomes for this patient population.

Another limitation of our systematic review is the small number of included studies, which limited our ability to assess publication bias. This limited number may be explained by the scarcity of RCTs involving patients with dyspnea and fatigue persisting for 12 weeks or more.

Key gaps identified include the absence of clinically meaningful thresholds for fatigue scales, the lack of assessor blinding in some of the included RCTs, and the limited investigation of multisystem mechanisms (e.g., inflammation), all of which outline priorities for future research. Additional gaps relate to the lack of standardization in the instruments used to assess dyspnea- and fatigue-related outcomes in PCC, which hinders meaningful comparisons across studies.

As clinical implications of this systematic review, we can highlight the following:

Isolated IMT appears insufficient for managing PCC symptoms, requiring combined strategies (e.g., MPT with psychological support). Given the prominent role of peripheral muscle deconditioning in individuals with PCC, participation in an MPT program aimed at improving muscle strength, physical functioning, and fatigue perception is essential. Rehabilitation protocols should account for the chronic and multisystemic nature of PCC, avoiding direct comparisons with acute COVID-19 or traditional respiratory diseases. The findings of this systematic review contribute to refining rehabilitation strategies for PCC, emphasizing muscle reconditioning over traditional cardiopulmonary metrics. It is also important to highlight that pulmonary rehabilitation, delivered either in-person or remotely, remains the standard physical intervention for improving dyspnea, physical function, and quality of life in patients with PCC [99].

## Conclusion

This systematic review and meta-analysis evaluated the effects of IMT and MPT in patients with PCC.

Regarding IMT, statistically and clinically significant improvements were observed in MIP, FVC, V̇O₂max, timed STS, and treatment adherence. Although FVC and the 1RM half squat percentage test showed statistically significant differences, their clinical relevance was low.

Regarding MPT, statistically and clinically significant improvements were observed in muscle strength as measured by the 30-s STS test, fatigue perception (FSS), and physical functioning. Upper limb strength, assessed using the HGS test, and lower limb strength, assessed using the TUG test, also showed statistically significant improvements, although their clinical relevance was low.

The certainty of evidence for all reported outcomes in both intervention groups (IMT and MPT) was rated as low. This indicates limited confidence that the estimated effects are close to the true effects, with some degree of variability remaining possible. Nonetheless, it is important to emphasize that the prioritized outcomes in this systematic review represent potentially meaningful benefits for the rehabilitation of patients with PCC.

Therefore, the findings of this review suggest that both IMT and MPT may provide clinically relevant benefits for patients with PCC, particularly in improving respiratory function and physical functioning; however, the certainty of evidence, as determined by the GRADE assessment, remains low. Further high-quality randomized controlled trials are recommended to strengthen these conclusions and guide evidence-based clinical decision-making.

### Differences between the protocol and the systematic review performed

The modifications made were:

1. Title, question, and purpose of the review
  The title of the review was modified because only one study applied the currently accepted diagnostic criteria for diagnosing ME/CFS, and no participants met the criteria.
2. Outcomes
  None.
3. Type of studies included
  None.
4. Schedule
  The expected completion date was January 2024, but the schedule was changed due to personal sickness in the family.

We consider that these modifications do not represent significant deviations from the protocol.

## Supplementary Information

Supplementary Material 1. Search strategy.

Supplementary Material 2. Excluded articles and reasons.

## Data Availability

The authors confirm that the data supporting the findings of this study are available within the article. Furthermore, the data sets used and/or analyzed during the current study are available from the corresponding author upon reasonable request.

## Abbreviations

ACSM: American College of Sports Medicine
BDI: Baseline Dyspnea Index
CFQ-11: Chalder fatigue scale
CI: Confidence interval
COPD: Chronic obstructive pulmonary disease
DeCS: Descriptors in Health Science
EQ-5D-3L: EuroQol-3D Questionnaire
EQ-5D-5L: EuroQol-5D Questionnaire
FEV_1_: Forced expiratory volume in 1 s
FVC: Forced vital capacity
GRADE: Grading of Recommendations Assessment, Development and Evaluation
HGS: Handgrip strength
I^2^: Higgins inconsistency test
ILD: Interstitial lung disease
IMT: Inspiratory muscle training
ITT: Intention to treat
K-BILD: King’s Brief Interstitial Lung Disease
MCID: Minimal clinically important difference
MD: Mean difference
Mesh: Medical Subject Headings
ME/CFS: Myalgic encephalomyelitis/chronic fatigue syndrome
MIP: Maximal inspiratory pressure
MR: Maximal repetition
mMRC: Modified Medical Research Council Dyspnea Scale
MPT: Multicomponent physical training
PCC: Post Covid conditions
PCFS: Post-COVID-19 Functional Status Scale
PICOS: Population, Intervention, Comparison, Outcome
PRISMA: Preferred Reporting Items for Systematic reviews and Meta-Analyses
PROSPERO: Prospective International Registry of Systematic Reviews
RCT: Randomized clinical trial
RoB: Risk of bias
SF-12: 12-Item Short-Form Health Survey
SF-36: 36-Item Short-Form Health Survey
SMD: Standard mean difference
STS: Sit-to-stand
5-STS: 5 Times sit-to-stand
TDI: Transition Dyspnea Index
TUG: Time Up and Go
$\dot{V}$ O_2max_: Maximal volume of oxygen
VO_2_peak: Peak consumption of O_2_
WHO: World Health Organization
