# Supplementary material for: Effectiveness of inspiratory muscle training and multicomponent physical training in patients with post-COVID conditions: a systematic review and meta-analysis
Source: Syst Rev. 2025 Nov 20;14:230. doi: 10.1186/s13643-025-02982-1 (PMC12636207; doi:10.1186/s13643-025-02982-1)
Supplement: Supplementary file 1 — Supplementary Material 1. Search strategy. [file 13643_2025_2982_MOESM1_ESM.docx]

**Supplementary Material 1.** Search strategy

| Cochrane Library  01 study | #1 MeSH descriptor: [Post-Acute COVID-19 Syndrome] explode all trees  #2 MeSH descriptor: [Physical and Rehabilitation Medicine] explode all trees |
| --- | --- |
| Embase  176 studies | (‘long COVID’/exp OR 'chronic COVID syndrome' OR 'chronic COVID-19' OR 'COVID long-hauler' OR 'COVID-19 long-hauler' OR 'long haul COVID' OR 'long haul COVID-19' OR 'long hauler COVID' OR 'post COVID 19 fatigue' OR 'post COVID 19 neurological syndrome' OR 'post COVID 19 syndrome' OR 'post COVID fatigue' OR 'post COVID impairment' OR 'post COVID syndrome' OR 'post-acute COVID syndrome' OR 'post-acute COVID-19' OR 'post-acute COVID-19 fatigue' OR 'post-acute COVID-19 neurological syndrome' OR 'post-acute COVID-19 syndrome' OR 'post-acute sequelae of SARS-CoV-2 infection' OR 'post-COVID condition' OR 'post-COVID-19 condition') AND ('rehabilitation'/exp OR 'functional readaptation' OR 'medical rehabilitation' OR 'readaption' OR 'readjustment' OR 'rehabilitation concept' OR 'rehabilitation engineering' OR 'rehabilitation potential' OR 'rehabilitation process' OR 'rehabilitation program' OR 'rehabilitation programme' OR 'rehabilitation, medical' OR 'rehabilitative treatment' OR 'resocialisation' OR 'resocialisation therapy' OR 'resocialization' OR 'resocialization therapy' OR 'revalidation' OR 'rehabilitation') |
| Epistemonikos  388 studies | Post-Acute COVID-19 Syndrome |
| PEDro  137 studies | COVID AND Fitness training OR COVID AND respiratory therapy OR  COVID AND strength training |
| PubMed/ MEDLINE  486 studies | ("Post-Acute COVID-19 Syndrome"[Mesh] OR (COVID-19 Syndrome, Post-Acute) OR (Post-Acute COVID-19 Syndromes) OR (Long Haul COVID-19) OR (COVID-19, Long Haul) OR (Long Haul COVID 19) OR (Long Haul COVID-19s) OR (Post Acute COVID-19 Syndrome) OR (Post Acute COVID 19 Syndrome) OR (Long COVID) OR (Post-Acute Sequelae of SARS-CoV-2 Infection) OR (Post Acute Sequelae of SARS CoV 2 Infection) OR (Post-COVID Conditions) OR (Post COVID Conditions) OR (Post-COVID Condition) OR (Long-Haul COVID) OR (COVID, Long-Haul) OR (Long Haul COVID) OR (Long-Haul COVIDs)) AND ("Rehabilitation"[Mesh] OR (Habilitation)) |
| Lilacs/VHL  323 studies | (mh:("Post-Acute COVID-19 Syndrome") OR (Síndrome Pós-COVID-19 Aguda) OR (Síndrome Post Agudo de COVID-19) OR mh:C01.748.610.763.500.500$ OR mh:C01.925.705.500.500 OR mh:C01.925.782.600.550.200.163.500$ OR mh:C08.381.677.807.500.500$ ​​OR mh:C08.730.610.763.500.500$ OR mh:C23.550.291.500.829.375$) |
| Web of Science  456 studies | ((long COVID* OR chronic COVID syndrome* OR chronic COVID-19* OR COVID long-hauler* OR post COVID 19 syndrome* OR post-acute COVID syndrome* OR post COVID 19 fatigue* OR post-acute sequelae of SARS-CoV-2 infection* OR post-acute COVID-19 neurological syndrome*) AND (Rehabilitation* OR Habilitation*)) |
| Scopus  55 studies | ("Post-Acute COVID-19 Syndrome"[Mesh] OR (COVID-19 Syndrome, Post-Acute) OR (Post-Acute COVID-19 Syndromes) OR (Long Haul COVID-19) OR (COVID-19, Long Haul) OR (Long Haul COVID 19) OR (Long Haul COVID-19s) OR (Post Acute COVID-19 Syndrome) OR (Post Acute COVID 19 Syndrome) OR (Long COVID) OR (Post-Acute Sequelae of SARS-CoV-2 Infection) OR (Post Acute Sequelae of SARS CoV 2 Infection) OR (Post-COVID Conditions) OR (Post COVID Conditions) OR (Post-COVID Condition) OR (Long-Haul COVID) OR (COVID, Long-Haul) OR (Long Haul COVID) OR (Long-Haul COVIDs)) AND ("Exercise Therapy"[Mesh] OR (Remedial Exercise) OR (Exercise, Remedial) OR (Exercises, Remedial) OR (Remedial Exercises) OR (Therapy, Exercise) OR (Exercise Therapies) OR (Therapies, Exercise) OR (Rehabilitation Exercise) OR (Exercise, Rehabilitation) OR (Exercises, Rehabilitation) OR (Rehabilitation Exercises)) |
| Medrxiv  18 studies | "Post-Acute COVID-19 Syndrome" |
| Opengrey  4 studies | "Post-Acute COVID-19 Syndrome" |
