# Supplementary material for: Effectiveness of inspiratory muscle training and multicomponent physical training in patients with post-COVID conditions: a systematic review and meta-analysis
Source: Syst Rev. 2025 Nov 20;14:230. doi: 10.1186/s13643-025-02982-1 (PMC12636207; doi:10.1186/s13643-025-02982-1)
Supplement: Supplementary file 2 — Supplementary Material 2. Excluded articles and reasons. [file 13643_2025_2982_MOESM2_ESM.docx]

**Supplementary Material 2.** Excluded articles and reasons

| **Reason** | **Study (Author and year)** |
| --- | --- |
| Missing data on the time of persistent symptoms | Da Silva et al. (2023) [34]; Li et al. (2022) [35]; Sharma et al. (2022) [36]; Ali et al. (2023) [37]; Arora et al. (2022) [38]; Teixeira Do Amaral et al. (2022) [39]; Mashhadi et al. (2022) [40] |
| Adult population restricted to a type of professional class | Hasenoehrl et al. (2022) [41] |
| Population with symptoms of fatigue and dyspnea for less than 12 weeks | Okan et al. (2022) [42]; Capin et al. (2022) [43]; Rodriguez-Blanco et al. (2023) [44]; Corna et al. (2022) [45]; Bagherzadeh-Rahmani et al. (2023) [46]; Tanhan et al. (2023) [47]; Şahın et al. (2022) [48] |
| RCT post-hoc | Metcalfe et al. (2023) [49] |
| Clinical trials protocols | Daynes et al. (2023) [50]; Gomes et al. (2023) [51]; Besnier et al. (2022) [52]; Turan et al. (2021) [53] |
| Congress summary | Karthikeyan, (2021) [54]; Del Corral et al. (2021) [55] |
| Wrong comparator | Altmann et al. (2023) [56]; Romanet et al. (2023) [57]; Espinoza-Bravo et al. (2023) [58]; Vallier et al. (2023) [59] |
| Studies whose design did not correspond to RCT | Smith et al. (2023) [60]; Lobanov et al. (2022) [61]; Putrino et al. (2021) [62] |
| Duplicate study excluded in the screening process | Albiach et al. (2023) [63] |
